# The sex‐selective impact of the Black Death and recurring plagues in the Southern Netherlands, 1349–1450

**DOI:** 10.1002/ajpa.23266

**Published:** 2017-06-15

**Authors:** Daniel R. Curtis, Joris Roosen

**Affiliations:** ^1^ Room 1.70, Doelensteeg 16, Leiden University, Institute for History Leiden 2311VL Netherlands; ^2^ Wittevrouwen 7bis, Utrecht University, Research Institute for History and Art History Utrecht 3512CS Netherlands

**Keywords:** black death, paleodemography, paleoepidemiology, selective mortality, sex

## Abstract

Although recent work has begun to establish that early modern plagues had selective mortality effects, it was generally accepted that the initial outbreak of Black Death in 1347‐52 was a “universal killer.” Recent bioarchaeological work, however, has argued that the Black Death was also selective with regard to age and pre‐plague health status. The issue of the Black Death's potential sex selectivity is less clear. Bioarchaeological research hypothesizes that sex‐selection in mortality was possible during the initial Black Death outbreak, and we present evidence from historical sources to test this notion.

**Objective:**

To determine whether the Black Death and recurring plagues in the period 1349–1450 had a sex‐selective mortality effect.

**Materials and Methods:**

We present a newly compiled database of mortality information taken from mortmain records in Hainaut, Belgium, in the period 1349–1450, which not only is an important new source of information on medieval mortality, but also allows for sex‐disaggregation.

**Results:**

We find that the Black Death period of 1349–51, as well as recurring plagues in the 100 years up to 1450, often had a sex‐selective effect—killing more women than in “non‐plague years.”

**Discussion:**

Although much research tends to suggest that men are more susceptible to a variety of diseases caused by bacteria, viruses and parasites, we cannot assume that the same direction of sex‐selection in mortality applied to diseases in the distant past such as Second Pandemic plagues. While the exact reasons for the sex‐selective effect of late‐medieval plague are unclear in the absence of further data, we suggest that simple inequities between the sexes in exposure to the disease may not have been a key driver.

## 1 INTRODUCTION

Recent literature has begun to establish that early modern plagues did not kill indiscriminately but had elements of selectivity in their mortality effects (Alfani & Murphy, 2017; Whittles & Didelot, 2016). For example, a number of studies over the years have suggested that from the fifteenth century onwards, plague acquired a “social character” with a preference for striking the poor (Carmichael, 1986; Cohn, 2010; Slack, 1985). However, this was not a unilinear process: the last great plagues of the seventeenth century in Italy returned to indiscriminately killing rich and poor alike (Alfani, 2013; Alfani & Murphy, 2017: 325–326). Other works have combined to suggest that recurring late‐medieval plague outbreaks killed children to a higher degree than adults (Galanaud & Galanaud, 2010; Höhl, 2002: 299; Razi, 1980: 134)—although not something that has been met with entire consensus (Carmichael, 1986: 90–3). This stands in contrast to the first plague of the Second Pandemic, the Black Death of 1347–52,^1^ which has been presented as a “universal killer” (Margerison & Knüsel, 2002; Naphy & Spicer, 2004)—likely testament to its high mortality rates. In recent years, however, bioarchaeological investigation has suggested that the Black Death may also have been selective in its mortality effects, particularly with regard to age and pre‐existing health status (DeWitte, 2010a; DeWitte & Hughes‐Morey, 2012; DeWitte & Wood, 2008).

The notion that plagues of the Second Pandemic selected by sex, however, has had far less confirmatory evidence. A few works have suggested that early modern plague led to more female deaths than male, mainly from those relying on documents (Ell, 1989; Frandsen, 2010: 358, 370; Pérez Moreda, 1987; Zapnik, 2007: 241; also including bioarchaeologists that have used documents; Signoli, Séguy, Biraben, & Dutour, 2002). By contrast, early pioneering work in a parish of early modern London suggested that male mortality outweighed female in the outbreaks of 1592–3, 1603 and 1625, citing differences in occupation and hygiene (Hollingsworth & Hollingsworth, 1971). More commonly, however, both older and more recent studies have generally suggested that the disease did not clearly discriminate by sex (Alexander, 1980: 258–9; Alfani & Murphy, 2017; Bradley, 1977; Höhl, 2002: 302; Jirková, 2015: 227; Schofield, 1977; Whittles & Didelot, 2016: 6). For the Black Death, bioarchaeological research has suggested much the same: the excavation of skeletons from a plague burial site (298 individuals) and a pre‐Black Death “normal” burial site (194 individuals) revealed no kind of sex‐discrepancy in mortality (DeWitte, 2009) (though the protocol used is not the same one applied in this article for calculating sex ratios in mortality). In a later publication, these findings were supplemented, however, with new evidence (DeWitte, 2010b). In the plague burial site, Black Death excess mortality was higher for males with osteological stress markers than females. Nevertheless, this could still suggest two different outcomes. On the one hand, the higher amount of burials of men with stress markers could mean that prior physiological stress increased the risk of death during the Black Death for men to a greater extent than women. On the other hand, lower excess mortality of women with stress markers may mean the Black Death was killing more otherwise healthy women than healthy men. More evidence is needed to confirm the most convincing interpretation.

There are a number of reasons why our knowledge in this field remains restricted. First, for the early modern plagues, the sample sizes are often not large enough to make strong claims. Sex ratios in mortality needs more data to reveal general patterns than studies of individual cities can provide, especially given that the differences in the ratios are often small. Second, for the medieval period, the amount of documentary source material to reveal mortality information, let alone sex‐disaggregated mortality information, is very limited. Many of the documents that medieval demographers have used relate only to male mortality or male heads of household—especially if they are reliant on fiscal or census sources. Even when such information is available, it tends to be for very restricted localities or time periods. Accordingly, research into the selectivity of plague in the medieval period has been dominated by bioarchaeologists. The pioneering work of Sharon DeWitte and associates has advanced our understanding of many facets of the medieval disease environment immensely. However, the third problem we identify is that some bioarchaeological approaches have potential accuracy issues with determining sex of skeletons. For example, DeWitte (2010b: 289) notes for the East Smithfield cemetery excavation that they determined sex from dimorphic features of the skull and pelvis, yet other studies “have demonstrated that the accuracy of these individual traits [in skull and pelvis], or combinations thereof, for the purpose of sex determination ranges from 68% to over 96%.” Potential inconsistencies in accuracy could be dangerous when one considers the limited samples only focused on one outbreak of plague in one burial site, and exacerbated when differences in sex ratios in mortality tend to be quite small anyway—although it must be noted that those recent studies using only reliable methods based on pelvic dimorphism (a >95% reliability in sex determination) have not revealed an excess of females in several plague cemeteries (Castex & Kacki, 2016). A fourth problem is understanding whether sex differentials in mortality during plagues were the result of inherent vulnerabilities to the disease itself, or instead caused by inequalities in exposure—a challenge in the absence of population figures (Carniel, 2008). For example, in one case in a series of plagues in Milan between 1452 and 1523, a higher mortality rate was noted for women, which was attributed to poor and overcrowded living conditions for female immigrants (Cohn & Alfani, 2007a). That is to say were more women dying here simply because more women lived here? This is a pressing point because most literature nowadays suggests that from the late Middle Ages onwards, urban environments tended to have more women, while rural ones had more men, a process sharpening in the transition to the early modern period (Bardsley, 2014; Kowaleski, 1999). This fourth problem then provides two separate challenges: first, we need to be able to separate rural and urban trends in sex ratios in mortality, and second we need to find long series of data to place plague mortality sex ratios within a broader context of “normal” sex ratios.

In this article we address all five challenges outlined above—small sample sizes, lack of medieval documentation, limitations to the bioarchaeological investigations, lack of systematic separation between urban and rural environments, and lack of chronological depth—to help us understand to a greater degree the potential sex selectivity of medieval plague. Building upon the pioneering bioarchaeological work of DeWitte, we offer a newly compiled database of documentary sources to follow up on the stimulating hypotheses she posed. Given the absolute scarcity of documentary evidence to furnish quantifiable indicators for mortality across large areas in much of the fourteenth‐ and fifteenth centuries (before the systematic recording of births, marriages and deaths in the parish registers from the mid‐sixteenth century onwards), we are fortunate to have a new dataset of 25,610 individuals from mortmain registers in Hainaut, a region of southern Belgium, across a long time period of 1349–1450.^2^ While bioarchaeological investigations have often had to use different cemeteries for pre‐Black Death and Black Death data (DeWitte, 2009; though skeletal series for the same city compared in Waldron, 2001), or compared archaeological data with attritional and catastrophic mortality (Castex & Kacki, 2016; Gowland & Chamberlain, 2005), this source allows us to look at the same localities over time (though unfortunately not for the pre‐plague period). The added value of this dataset is that the mortality information allows for specifically sex‐disaggregated data—highly unusual for late‐medieval Europe. Furthermore, as we go on to show later in the article, the recording process seemed to offer no intrinsic biases with regard to sex of the dead—that is to say, the source does not appear to have structurally discriminated against the recording of women.

## 2 MATERIAL AND METHODS

This section is divided into two. First, we discuss the source, its potential and limitations, and how we employ it for the purposes of revealing more about the potential sex‐selective nature of the Black Death and recurring plagues up to 1450. Second, we discuss how we identify the plagues—the Black Death may be obvious, but for the recurring plagues it is not straightforward.

### 2.1 Employment of the mortmain database

Mortmain accounts are similar to heriot taxes found in England in two distinct ways. First, they were paid as a death duty, and second, payment was usually in the form of the best movable possession of the deceased. However, unlike the mortmain, heriots were levied on tenant holdings and not on individuals. Accordingly, heriots indicate the death of the head of a household (Arthur, 2010: 60), usually older and male, not only leading to an overall underestimation of mortality (Benedictow, 2004: 375), but also problematic for the purposes of calculating sex‐selection in mortality. Previous scholars confused the Hainaut mortmain records for heriots, which can explain why those that made use of the source in the past failed to record more than the (presumed) head of household in a line (Blockmans, 1980; Sivéry, 1965). Mortmain was not just paid by tenant heads of households but by a large group of people coming from a lineage of servitude who now enjoyed free status (Verriest, 1910: 248–50; and for nearby Flanders; Kittell, 2013). It is important to note that the mortmain did not just record women “heads of household” who were widowed, but recorded women who had died even when their husband was still alive, and furthermore, when women were widowed their status was specifically mentioned as such, which contrasted with other indications of marital status such as “wife of *x*” (ADN B 12178, fo.5r). This guards against a view that women found in the mortmain may have been at a disadvantage compared to men (on the relatively poorer position of single females in medieval and early modern Europe; Bennett & Froide, 1998; a more positive view in Devos, De Groot, & Schmidt, 2015), and removes a potential selection bias in the data.

Overall, from the whole database across the period 1349–1450, there were 25,610 deaths recorded. The vast majority of these names were adults and subadults—that is to say the mortmain does not systematically record children. This is because the mortmain applied to all men and women who had reached “age of majority,” and in the Southern Netherlands this was generally quite high—anything between 18 and 25 years (Howell, 1998: 108; Verriest, 1910: 305–6). A proportion of the database (1.8%) were not identifiable as male or female (and thus excluded from the calculation of sex ratios in mortality), and given that they often came with the note “child of *x*,” it would be reasonable to assume that they were the few children that managed to appear in the record. Even these were not all children, however, but often simply adults or subadults still living in the same household as their parents and explicitly indicated as being of legal age to purchase the impounded goods from their deceased parent/s (ADN B 12178, fo. 6v). Overall then, we know that 98.2% were almost definitely adults or subadults. Accordingly, the mortmain can only be used to assess the sex‐selective effect of plagues on adult and subadult mortality. Although it would be preferential to separate sex‐selective mortality into various age brackets, since some previous works have suggested that the sex ratio of plague victims varied according to age (Hollingsworth & Hollingsworth, 1971; Ell, 1989; other works did not find this—for a synthesis; Alfani & Murphy, 2017: 323–4), the mortmain does not provide us with the exact ages of the dead.

If we take the whole database from 1349 to 1450 we acquire an average sex ratio in mortality of 1.25:1.^3^ However, some select localities had special stipulations leading to an under‐assessment of women in the database. Maubeuge was removed because women with children were not included in this city's assessment (Verriest, 1910: 309), and this decision is corroborated by its average sex ratio in mortality of 4.31:1—quite anomalous to the rest of the database. A few rural localities were also excluded from the overall database, for they deviated from the general rule of including all men and women (as described above), and had a bias towards men for a specific reason (Verriest, 1910: 308).^4^ An example of one of these locally‐specific customs was that if the wife died first, no mortmain was to be paid in the village of Froidchapelle‐Fourbechies (and thus no record of her death) (Verriest, 1910: 308). After the exclusion of all localities with female‐specific exemptions, the number of individuals in the mortmain database was 22,247, and this produced an overall average sex ratio for the period 1349–1450 of 1.07:1. Still, even this figure may reflect a certain degree of female exclusion, but ultimately the value of this source is not in the absolute figures it provides, but in the relative figures—essentially how the sex ratio in mortality changes over time and how it can be systematically compared between plague and “nonplague” years.

Aside from recording women to a much fuller degree than other contemporaneous documentary sources such as the heriots, another quality of this database is that men and women can be accurately identified using a multitude of approaches. We attribute female status to a recorded name on the basis of (a) first names, (b) kinship ties where it was mentioned that an individual was the wife, mother, daughter or sister of *x*, (c) impounded goods were sold back to the husband of the individual that died (providing further proof we are not dealing with only widows), (d) goods were sold to “*a se baron*” (only mentioned for women, and likely referring to a form of male patronage), and (e) mention of “*se meskine*” (referring to female social status and/or profession; Bourguignon & Dauven, 2012).

Of course, there are some limitations to the source—some very general ones characteristic of many medieval documents such as delayed or incomplete registration during political strife and conflict, although their value is that mortality trends can be compared in known “crisis” years (such as known plagues) with “normal” or “noncrisis” years (Thoen & Devos, 1999: 128). However, mortmain cannot be viewed as a “direct” indicator of mortality, since it could also increase or decrease depending on differential enforcement of the rights of the overlord to extract possessions after death. In nearby Flanders, the Count was increasingly able to subject more people to mortmain payment in the late fourteenth century: it was only after 1380 that people were recorded in a standardized way, the rubric of the document itself began to change, and explicit reference to the precise item seized occurred (Kittell, 2013: 192). Fortunately, this does not appear to have been the case for Hainaut though as the structure and terminology of the documents did not change from the first extant document of 1349 all the way up to 1500, indicating a standardized procedure in place from the start. Moreover, there do not seem to be any structural increases in the amount of people recorded that are not linked to a period of high mortality identified in the literature. The issue here instead was that the number of districts (and therefore localities) included in the account showed some fluctuations over time. Indeed, certain cities were able to secure freedoms from assessment. Native burghers of Ath were exempt from 1284 (at the latest), in Mons in 1295, in Valenciennes at an unidentified date, in Binche in 1265, in Beaumont (only the fortified area) in 1383, in Soignies (only the franchise) in 1142, and in Le Roeulx at an unidentified date (Verriest, 1910: 323–33). Many of these cities still appeared in the assessment, however, since the privileges did not apply to “nonburghers” or recent immigrants—in fact only Valenciennes failed to appear at all. However, these issues relate to calculation of overall plague mortality rates, rather than substantially distorting relative male‐female mortality ratios. The exemptions for native burghers of the aforementioned cities applied equally to men and women, and were in place well before the first extant record with the exception of Beaumont. In any case, we try to cater for this issue by also testing a set of localities across a shorter timespan that are almost ever‐present, in addition to the overall results from the whole database (see pp. 15–16).

### 2.2 Identifying plagues

The reason why many bioarchaeological studies have focused on the initial Black Death outbreak rather than any other recurring outbreak of medieval plague is because almost all places in Western Europe experienced it (reaching Southern Europe earlier than the North). There can be little contention then about whether plague occurred in these years or not. In fact, this issue has been settled in recent years with the various confirmatory findings of the causal bacterial agent *Yersinia pestis* in various mid‐fourteenth‐century burial sites (the different studies described in Benedictow, 2016: 81–2; Little, 2011). Indeed, one of the strengths of bioarchaeological research into the East Smithfield burial site in London is that it was founded in 1349 explicitly in anticipation of having to bury the plague dead—thus allowing control for cause of death to a greater extent than other mortality samples with many different causes of death (DeWitte, 2010b: 290).

**Figure 1:**
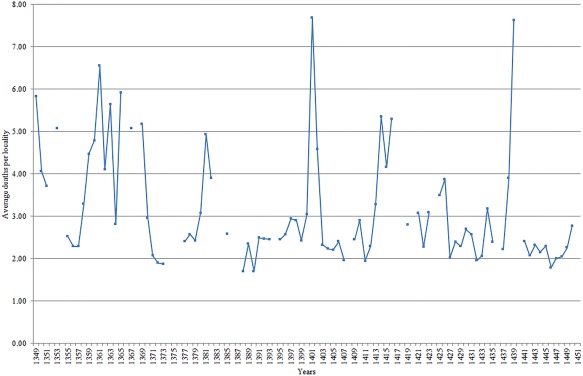
Annual mortality based on individuals owing mortmain, Hainaut, 1349–1450 (average deaths per locality)

In our mortmain database, the very first extant record fortunately comes from 1349 and so we can cover the Black Death period up to May 1352, after which the mortality peak subsided. It must be noted that the record begins in June 1349, and thus there is a possibility that it may already have missed some plague deaths by this time. However, indications from sources across the Southern Netherlands such as pressures on existing cemeteries, outbreaks of Jewish persecution, flagellant movements, and so on suggest that the Black Death only began to reach its height from the summer of 1349 onwards (see our Supporting Information Appendix 1 for more details). There is no evidence that the Black Death had reached Hainaut by 1347–8 as it had done in the Mediterranean. In fact, it is said to have arrived in Valenciennes in June 1349, followed by Tournai, Mons, and Lille in early July (Benedictow, 2004: 113; Sivéry, 1965: 438). Considering the timing of the Black Death in Hainaut, it is likely that the first extant mortmain account starting on 24 June 1349 was constructed as a direct consequence of hyper‐mortality—and thus we can be confident that we are able to capture the major part of the Black Death period. However, it must be acknowledged at least that the mortmain account available for Hainaut offers an absolute minimum assessment with regard to the mortality effect of the Black Death, as shown by Figure 1 where the mortality spike for the Black Death was still smaller than some recurring plagues. This is down to some issues with the Hainaut mortmain accounts. First, the initial account for 24 June 1349–11 April 1350 only covers a period of 10 months, while other documents cover a full 12 months. Second, there was some disruption in local administration due to the death of local bailiffs: during the Black Death period we see counts having to be performed by different local officials, which is something that does not happen in any other year (NAB, I, 004, 17867). Third, since the first extant document was triggered by hyper‐mortality, local administration was likely caught off guard during the very initial stages of the epidemic. Fourth, the first manuscripts of the mortmain are in a much worse state than the manuscripts that follow—possibly extending the missing information to an estimated 15–20% on account of the size of missing sections and average number of individuals recorded per folio. Fifth, we must also take into account the notion that many of the recurring plagues seen in the late‐medieval Southern Netherlands were also very severe (Sivéry, 1966; Thoen & Devos, 1999).

We are also fortunate to have a long run of accounts, notwithstanding a few missing years, for a period of 102 years up to 1450 where we can examine the sex‐selective effects of recurring plagues too. This is important given too much research at present is polarized between the initial Black Death outbreak on the one hand, and the early modern plagues of the sixteenth‐ and seventeenth centuries (likely due to the increasing appearance of documentary source material that can be employed quantifiably). Indeed, it has been recently remarked that “we know little about the outbreaks in the century immediately after the Black Death” (Alfani & Murphy, 2017: 318)—that is to say the recurring outbreaks of the late fourteenth‐ and fifteenth centuries.

One issue, however, is how we identify which years from 1349 to 1450 were plague years. As well as the aforementioned DNA evidence of *Yersinia pestis* for the Black Death, there is good evidence of this pathogen as causal agent for the second plague outbreak of 1358–63 in the Southern Netherlands. Recent evidence has overturned the initial identification of *Yersinia pestis* DNA at Bergen‐op‐Zoom as the specific Black Death strain (which was mistakenly dated to 1349/50; Haensch et al., 2010), instead identifying it as the same strain present in the “*pestis secunda*” outbreak in London (London *Yersinia pestis* genome 6330) (Bos et al., 2011; Green and Schmid, 2016; Spyrou et al., 2016). Nonetheless, due to a lack of studies identifying the bacterial agents in other recurring plague outbreaks, current research often has to rely on those years which are said to have been “plague‐years” in the historiography, accepting the disease diagnosis at face‐value. This is problematic since according to some scholars, the paucity of sources and the considerable gaps exhibited, often make it impossible to separate late‐medieval plague from other diseases and causes of mortality (Thoen & Devos, 1999). However, we propose a threefold approach which can help identify likely plague years, even if we have to acknowledge that the only period in which we can be reasonably sure that epidemic mortality was almost wholly attributable to plague was the initial Black Death. In the first stage of our approach we isolate years where there were unusual mortality spikes compared to normal years. This can be done on the basis of the mortmain record itself, and thus represents an indicator of ‘possible plague years’. In Figure 1 below, plague years could possibly have occurred in the mortality spikes (those years with an average rate of mortalities per locality of more than three), 1349–51, 1353, 1358–63, 1365, 1367–9, 1380–2, 1400–2, 1413–16, 1421, 1423, 1425–6, 1434, and 1438–9.^5^ Given the issue of the changing territorial reach of administration of the mortmain record (more localities could be included in some years); an average rate of deaths per locality mentioned in the record is preferred to simply calculating the spikes by aggregate numbers of mortmain dead.

Once we know the main dates in which mortality was higher than normal, our second method is to compare these dates with a new database of “explicit plague mentions in archival sources” (see Supporting Information Appendix 1). A similar enterprise was undertaken by Biraben (1976) on a pan‐European level, which has recently been subject to some high‐profile digitization (Büntgen, Ginzler, Esper, Tegel, & McMichael, 2012: 1587; Christakos, Yu, Wang, Serre, & Olea, 2005: 202–3; Schmid et al., 2015; Voth & Voigtländer, 2013; Yue, Lee, & Wu, 2016). However, this original pioneering work inevitably suffered from geographical disparities in scholarly attention, presenting France as the epicenter of all Second Pandemic plagues, while the Low Countries experienced virtually no plagues across a period of 400 years. Our database is also an improvement in distinguishing between different “types” of source reference, using a three‐category division of quantifiable, contemporary description, and later histories or memories (proposed originally by Blockmans, 1980). Naturally, caution should be shown with this kind of exercise, since many of the works indicating a plague in a particular year in the medieval period do not provide absolute unequivocal evidence for plague per se, but either explicitly point to a disease of some kind, or give an indication of a mortality crisis that may have been caused by plague. Only from the second half of the fifteenth century do we begin to see explicit differentiation in the diseases from the Low Countries sources: a good early example are the references for the epidemic in the Duchy of Guelders in 1472–3 and 1497–8, which are described as “*rode loop*” and “*rood melisoen*” (dysentery) and “*pokken*” (smallpox) respectively (Schaîk, 1987: 305–6). However, we are helped by escaping localism and establishing a broader geographical and temporal view. The term “*peste*” found in one locality in 1 year may be rather weak evidence, if it were not for other localities also mentioning “*peste*” in the same year. Furthermore, the medieval Low Countries sources often produced a number of other terms to more explicitly refer to the plague: “*haestige sieckte*” (rapid sickness) referred to the disease's key feature which was the short period between visible symptoms and imminent death (Cohn, 2008). Other frequent terms included the “*heete siecte*” (hot sickness) and the “*heete ongemac*” (hot discomfort), in reference to the burning feverous state that the afflicted experienced, as well as the “*gave gods*” or the “*gods vandinge*” (the gift from God), in reference to the providential explanation of the disease's origins.

Overall, by matching the years with mortality spikes with the years found in our database of plague mentions in the sources for the medieval Low Countries, we suggest that plague outbreaks occurred in 1349–51, 1358–63, 1368–9, 1380–2, 1400–2, 1413–16, 1425–6 and 1438–9, even if we have to accept that other mortality causes occurred around or simultaneous to these outbreaks. For other raised mortality years in 1353, 1364–7, 1421, 1423, and 1434, there was no specific documentary evidence to point to likely plague outbreaks, even if the presence of the disease cannot be entirely ruled out.^6^ Many of these mortality spikes instead were likely down to famine or famine‐related diseases judged on the evidence of contemporary commentators from the Southern Netherlands and neighboring German regions citing, for example, incessant rainfall and scarcity in 1366 (Pertz, 1841: 35), a combination of storms, cold weather and rain in the period 1421–3 (Easton, 1928: 77) (leading to numerous flood events; Buisman, 1996: 446), ‘horribly severe cold’ and ‘terrible frost’ in 1434 (Despars, 1839: 337) and storms across the whole of the Low Countries (Gottschalk, 1975: 142). An outbreak of “*blarenpest*” (smallpox) was cited in nearby Limburg for 1367 (Rutten, 1997: 32).

As a third and final stage in the process, we also subject these identified plague years between 1349 and 1450 to an analysis of household clustering of deaths. One of the hallmarks of Second Pandemic plagues it has been suggested is the frequent death of those in close proximity to one another. This has led to the suggestion that plague may have been contagious—passed not only between humans via a vector, but even passed directly from human‐human interaction (Ayyadurai, Sebbane, Raoult, & Drancourt, 2010; Cohn, 2008; Hufthammer & Walløe, 2013; Walløe, 2008: 71; Welford & Bossak, 2010). Of course, this is a highly debatable and unresolved topic, but what is less debatable is the basic historical evidence that humans in close proximity were dying in greater numbers during plagues than in normal times (Alfani & Murphy, 2017; Cohn & Alfani, 2007b; Whittles & Didelot, 2016; although all of this evidence is from the early modern period). It must be noted, however, that this household clustering of mortality cannot be invoked on its own as evidence for the presence of plague. Indeed, many contagious diseases or other diseases connected to insalubrious or unhygienic conditions, for example, may have produced similar clustering of deaths. The idea here is simply to show that any noticeable increase of household‐clustered mortality is a further indication that a mortality peak was caused by the presence of an infectious disease.

**Figure 2:**
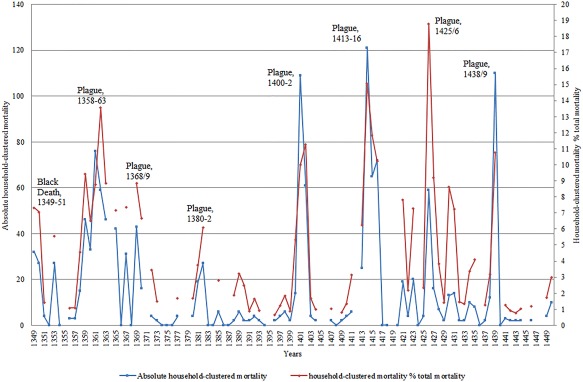
Household‐clustered mortality patterns, Hainaut, 1349–1450

As soon as two or more members of the same family died in a given year, they were linked together through their kinship tie in the mortmain database. Here, given the limitations of the medieval sources, we have to make an assumption that the linked kin were actually resident within the same physical household—which of course was not always the case. Figure 2 shows both the absolute number of instances where multiple members of the same household were dying as well as a percentage of total mortality these multiple household deaths represented.^7^ To calculate this, we counted, for example, a household with three dead members as three individuals and a household with five dead members as five individuals—therefore coming to a total of eight individuals dying within a household clustering rather than two instances. This was done to account for different sizes of households and to allow for calculation of the proportion of clustered deaths within the total mortality figures. Overall, Figure 2 indicates an association between multiple family members dying and the suggested “plague‐years” we have already identified above, lending further credence to our view, even if this cannot be seen as outright absolute confirmatory evidence.

## 3 RESULTS

**Figure 3:**
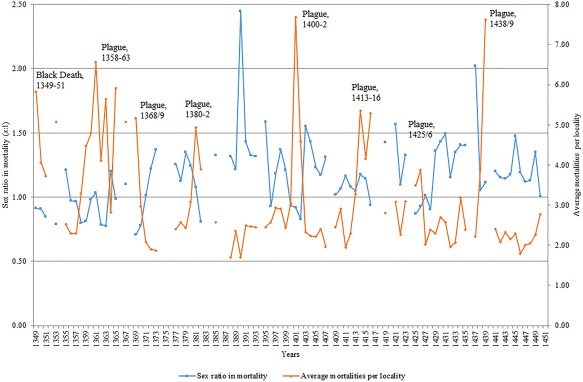
Sex ratios in mortality against overall mortality levels, Hainaut, 1349–1450

Figure 3 below shows the main plague periods from 1349 to 1450, the relative mortality spikes they caused, and this time compared with the sex ratios in mortality. Ultimately, our main finding is that when a major plague occurs, there is generally an inverse relationship with the sex ratio in mortality: that is to say it often correlates with a decline in the sex ratio, suggesting that more women were dying during plagues than in normal periods. This case could be made for the identified plague periods of 1349–51, 1358–63, 1368–9, 1380–2, 1400–2 and 1425–6, to a lesser extent in 1438–9, but is not strongly discernible for 1413–16. That the plague years 1413–16 and 1438–9 did not conform to trends of the other plague years is perhaps less surprising given these two periods were also characterized by upsurges in conflict in the southerly parts of the Southern Netherlands (Bocquet, 1969: 51), and thus the increased presence and death of soldiers may have led to some excess male mortality—especially since 1438–9 was also simultaneous to a serious famine (Curtis, Dijkman, Lambrecht, & Vanhaute, 2017; Derville, 1999: 215; Verhulst, 1963: 70) linked to the exceptionally cold decade of the 1430s (Camenisch, 2015). Recent research into differential spatial concentration of mortality in the city of Dijon in Eastern France has suggested that while the 1400–1 epidemic was likely plague, raised mortality in 1438–40 may in fact have a “different, possibly waterborne, disease involved,” even if plague co‐existed simultaneously (Galanaud, Galanaud, & Giraudoux, 2015). Overall, for such a large series of data (102 individual years), a negative Pearson correlation coefficient of −0.47 for mortality against sex ratios in mortality is strong, though this negative correlation may have been even stronger given that raised mortality in some years had causes other than plague and thus was not necessarily correlated with a lower sex ratio. The famine of 1390, for example, was correlated with a raised sex ratio—in line with expectations of a “female mortality advantage” during periods of severe malnutrition (Macintyre, 2002). That proportionately more women were dying during the main plague years than in “normal” years can be further demonstrated from Table 1 below. There is a gap of 0.24 between the sex ratios in mortality calculated for plague and nonplague years, and an extremely statistically significant association between outcomes (male/female deaths) and groupings (plague/non‐plague years). Furthermore, the results show that the Black Death did not differ in its sex‐selective effect from other recurring plague outbreaks, as evidenced by the insignificant statistical association.

**Table 1:** Sex ratios in mortality, Hainaut, 1349–1450

	Total	Male	Female	Unknown	Sex ratio
All years	22,247	11,292	10,559	396	1.07:1
Just plague years	9,213	4,332	4,634	247	0.94:1
Years without plague	13,034	6,960	5,925	149	1.18:1
Fisher's exact test		*p* value <0.0001			
Only Black Death	865	342	383	140	0.89:1
Only recurring plagues	8,348	3,990	4,251	107	0.94:1
Fisher's exact test		*p* value 0.5352			

Source: see fn. 2.

Notes: A large *p* value (>0.05) indicates weak evidence against the null hypothesis, thus we do not reject the null hypothesis. A small *p* value (< 0.05) indicates strong evidence against the null hypothesis, thus we reject the null hypothesis. This is applicable for Tables 1, 2, 3, 4.

As mentioned in Section 2, there is an issue that some localities disappear from the record and then reemerge—put simply, not all localities are ever‐present across the whole 102 years of the mortmain record we analyze. Often this refers to small villages or hamlets that normally record only one or two deaths anyway, and thus we do not expect this to significantly shape the results. To be more certain of this, however, we have decided to take a sample of localities that are almost ever‐present across a much shorter time period. In Table 2, therefore, we also have a sample of 61 localities that reappear in at least 8 of the 13 years between 1395 and 1407.^8^ We chose this sample period on the basis of (a) it includes an almost definitely identifiable plague period (1400–2), (b) this plague period is one of the strongest to hit Hainaut in the late Middle Ages, and (c) the period 1395–1407 is one of the few times where there is a clear run of years without any gaps. As Table 2 shows, even when looking at a shorter period with a sample of mainly present localities, the same phenomenon described above in Table 1 and Figure 3 is seen—a reduced sex ratio in mortality during the plague years when compared to nonplague years—and this time by an even clearer degree. We can be more confident then that the disappearance and reappearance of certain localities in the database is not driving the results we identify.

**Table 2:** Sex ratios in mortality, select sample in Hainaut, 1395–1407.

	Total	Male	Female	Unknown	Sex ratio
All years	2,489	1,298	1,183	8	1.10:1
Just plague of 1400/2	890	414	470	6	0.88:1
Years without plague	1599	884	713	2	1.24:1
Fisher's exact test		*p* value <0.0001			

Source: see fn. 2.

**Figure 4:**
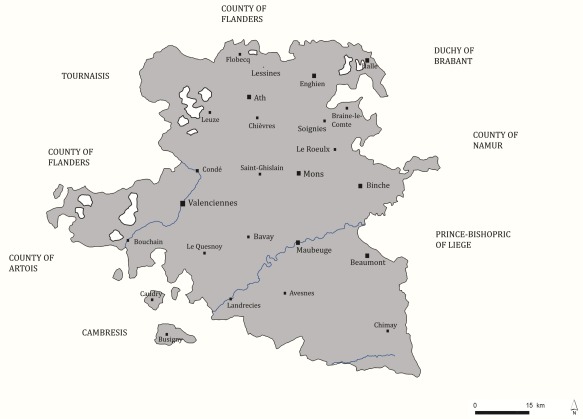
Map of Hainaut with urban localities in mortmain database

That late‐medieval plagues were associated with divergences from the norm in the sexual distribution of mortality is clear. However, we need to determine whether this was down to the intrinsic differences in resistance to the disease between the sexes, or instead a matter of differential male‐female exposure to the disease. For example, scholars have suggested that from the late Middle Ages onwards more women than men began to live in the cities, and this is significant when considered in tandem with the assumption that plagues were more severe in the cities than the countryside (Bardsley, 2014; Kowaleski, 1999). We review this assumption by systematically separating urban environments from rural. Although accurate population indicators for individual localities are scarce for the late‐medieval Southern Netherlands, it is known that the so‐called “*bonnes villes*” (richest localities that were usually fortified) were not subject to the fiscal hearth count (Arnould, 1956: 67–8). Hearth counts were only conducted in the countryside, and thus any locality in the mortmain found in the hearth count can be considered rural. The localities classified as urban in the mortmain record are labeled on the map of the County of Hainaut below (Figure 4)—only the city of Valenciennes never appeared in the mortmain.

Our results from Table 3 below suggest that the differential exposure hypothesis had some role to play. The decrease in sex ratio in mortality for plague years when compared to nonplague years was larger in the cities than in the countryside—0.31 in the cities compared to 0.22 in the countryside. However, even if it was not as sharp, plagues still led in the countryside to heightened female mortality relative to male compared to the norm: the phenomenon was seen regardless of urban or rural environment. In both urban and rural environments the association between outcome (male/female deaths) and grouping (plague/non‐plague years) was extremely statistically significant, and just like with the overall results in Table 1, the Black Death showed similar sex‐selective effects to the other recurring plagues in both city and countryside, as evidenced by the insignificant statistical association. Indeed, we would not expect a strong skew of women towards the cities in late‐medieval Hainaut anyway, given that this southerly region did not have the kind of urban network able to consistently attract steady streams of migrants—a small scattering of aforementioned towns but nothing to compare to the dense patchwork of powerful cities in Flanders, for example (Blockmans, Pieters, Prevenier, & Schaîk, 1982: 46).

**Table 3:** Urban and rural sex ratios in mortality, Hainaut, 1349–1450

	Urban				
	Total	Male	Female	Unknown	Sex ratio
All years	5,188	2,620	2,483	85	1.06:1
Just plague years	2,183	999	1,126	58	0.89:1
Years without plague	3,005	1,621	1,357	27	1.20:1
Fisher's exact test		*p* value <0.0001			
Only Black Death	223	84	101	38	0.83:1
Only recurring plagues	1,960	915	1,025	20	0.89:1
Fisher's exact test		*p* value 0.7			
	**Rural**				
All years	17,059	8,672	8,076	311	1.07:1
Just plague years	7,030	3,333	3,508	189	0.95:1
Years without plague	10,029	5,339	4,568	122	1.17:1
Fisher's exact test		*p* value <0.0001			
Only Black Death	642	258	282	102	0.92:1
Only recurring plagues	6,388	3,075	3,226	87	0.95:1
Fisher's exact test		*p* value 0.654			

Sources: see fn. 2.

Although we suggest in the Hainaut case that lower sex ratios in mortality during plagues was unlikely to have been only a case of more women resident in the cities, the differential exposure hypothesis could have applied in other ways, however. For example, on the micro level of the household, it may have the case that women were more likely to be in physical positions conducive to the spread of plague. Given that as already mentioned, recent literature has asserted the possibility that plague could have spread human‐human (whether through a vector or directly), afflicting people at close quarters (seen in household patterns of infection), a case could be made for the effects of women spending disproportionate amounts of time in houses and attributed a ‘care‐giving’ role within their own family (Rawcliffe, 1995: 182–3).

**Figure 5:**
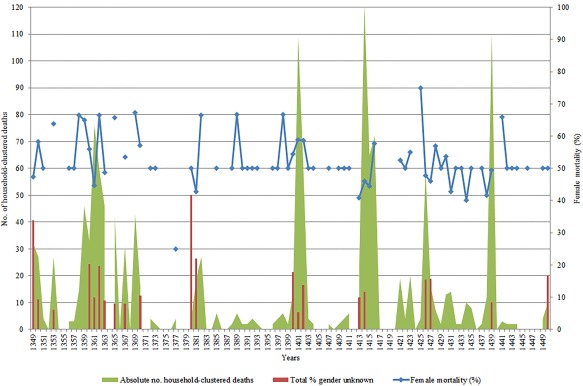
Female mortality in household clusters (%), Hainaut, 1349–1450

The mortmain database gives us a rare opportunity to explore this hypothesis further, by only focusing on those mortalities clustered in households—that is to say only including those cases where two or more people from the same (assumed) household have died (as reconstructed already in Figure 2). Only those cases have been included in which we know the sex of all household members—therefore a number of cases have been omitted and indicated as a percentage of sex ‘unknown’. In plague years this was a higher amount than in non‐plague years—11% compared to 4%—and the high amount in the Black Death period in particular was simply down to the greater damage to the manuscript itself which prevented accurate gendering of more individuals in the mortmain. After isolating only household‐clustered mortality, what can be discerned from the results is that the increased mortality of women vis‐à‐vis men during plagues compared to non‐plague years seen in Tables 1, 2, 3 did not appear to be driven by spatial inequalities between the sexes within the household. Indeed, Table 4 below shows that for specifically deaths occurring in clusters of two or more, the sex ratio in mortality was lower in plague years than non‐plague years but the difference was small—0.85:1 compared to 0.88:1. While in some of the plague years in Figure 5, females dominated the household clustering of mortality such as in 1358–63 or in 1425–6, there were clearly many other identified plague years when this was not the case. Indeed, there was no overall statistical significance in association between outcomes (male/female deaths) and groupings (plague/non‐plague years), with the Black Death behaving in the same way as the other recurring plagues. Ultimately what we can infer from these results is that either (a) women were not spending more time than men in houses or offering care‐giving roles, contrary to our assumptions, or (b) women were doing so, but this was not an important driver of overall increased female susceptibility to the disease, or (c) our reconstruction of household clustering, where sources do not allow for identification of household members spared by the plague (therefore making it difficult to reconstruct exactly the sex structure of the household and to measure differential risks of infection) might not be of a level needed to detect the effects of care‐giving or spending time in houses.

**Table 4:** Sex ratios in household‐clustered mortality, Hainaut, 1349–1450

	Total	Male	Female	Unknown	Sex ratio
All years	1,417	592	688	137	0.86:1
Just plague years	1,024	419	492	113	0.85:1
Years without plague	393	173	196	24	0.88:1
Fisher's exact test		*p* value 0.8046			
Only Black Death	63	22	25	16	0.88:1
Only recurring plagues	961	397	467	97	0.85:1
Fisher's exact test		*p* value 1			

Sources: see fn. 2.

## 4 DISCUSSION

We suggest that women died in greater proportions during plagues compared to normal times, and is something seen in Hainaut, Belgium, during the initial Black Death but also frequently throughout many recurring late‐medieval plague outbreaks up to 1450 suggesting this feature may have persisted over the long term. Of course, we must take into account the fact that although we know the overwhelming majority of the data to represent adult and sub‐adults, we cannot disaggregate these results further into precise age brackets—and so we cannot be sure whether a particular adult age‐group is driving these results.

Although we considered the possibility that this could have reflected simply an inequality in exposure to the disease, the evidence does not support this—in as far as we can feasibly test for it. Overall we show that the trend of a lowering in the sex ratio in mortality during plagues cannot be wholly put down to more women resident in cities since it was a phenomenon clearly discernible for both urban and rural environments. Furthermore, evidence from the clustering of deaths in households suggests that the likelihood of women spending more time in houses in domestic or care‐giving roles was also not likely a key driver in this trend, although we should be cautious on this second point: we still know relatively little about the actual environment in which the plague victims lived. We should also clarify that the results have been taken from one region only, and thus it remains to be seen if comparable data from the same or different periods in other parts of Europe would corroborate our findings.

By not supporting an interpretation based on differential exposure, this gives some credence to the possibility that the intrinsic interaction with the disease and differential male‐female resistance to plague played a role in the skewed sex ratios in mortality. If this was indeed the case, this is in contrast to a number of other diseases caused by bacteria, viruses, fungi, and parasites seen in the modern period (Blondiaux, Broucker, Colard, Haque, & Naji, 2015; Falafas, Vardakas, & Mourtzoukou, 2008: 627; Jansen, Stark, Schneider, & Schoneberg, 2007; May, 2007; Mehraj, 2004; Owens, 2002), which shows that we cannot assume that the pre‐industrial epidemiological experience should necessarily mirror the modern. Indeed, it is also known that other diseases such as malaria and chronic obstructive pulmonary disease disproportionately affect women (Cote & Chapman, 2009). Furthermore, if we accept an “intrinsic” rather than “exposure” explanation, we differ from much literature on modern forms of plague that assert differences in behavior. So plague mortality is said to be skewed towards men in the United States through hunting and ranching activities (Butler, 1989; Poland, 1989), with increased risks of engaging directly with infected animal vectors (Cleri et al., 1997; Perry & Fetherston, 1997). Elsewhere, in Lushoto, north‐eastern Tanzania, women are said to be more commonly infected on account of sleeping on the floor (men sleep in beds) (Davis, Makundi, Machang'u, & Leirs, 2006; Kamugisha et al., 2007). Overall, we must remain cautious in the absence of more confirmatory data from other places and periods, and that we still know relatively little about the daily behavior of men and women in the medieval period.

## 5 CONCLUSION

Recent scholarship has increasingly suggested that early modern plagues were (a) not all the same in their epidemiological characteristics (Alfani, 2013; Curtis, 2016), and (b) may have been selective in some respects (Alfani & Murphy, 2017), even if this was not a straight uni‐linear process from a less selective to a more selective disease (Alfani, 2013). This contrasts with the Black Death of the mid‐fourteenth century which has often been portrayed as a “universal disease” (Naphy & Spicer, 2004), testament to its great severity, which disrespected age, sex, health, and socio‐economic position. Only in recent bioarchaeological work has evidence been put forward that suggests a level of selection was discernible with the Black Death too on the grounds of age and frailty (DeWitte, 2010a; DeWitte & Hughes‐Morey, 2012; DeWitte & Wood, 2008), while on the grounds of sex it was, at the very least, not ruled out either (DeWitte, 2010b).

This article lends support to the notion that the initial Black Death does not necessarily deserve to be considered as “anomalous” or “separate” to other outbreaks that followed in its wake, and may have displayed at least some similar features—and specifically the capacity to be sex selective. This is the first time that an effort has been made to compare a characteristic of the Black Death such as sex selectivity to other recurring plagues and to other “nonplague years” in the same (large) region over a long (early) period using the same (fairly standardized) source. Our main finding is that the sex ratio in mortality tended to decrease during the Black Death and recurring plagues in comparison with non‐plague years, and by comparing urban and rural sex ratios in mortality, as well as mortality clusters within households, we suggest that this was not necessarily driven by differential exposure to the disease between the sexes.

## ACKNOWLEDGMENTS

This article has been funded by the NWO in the Innovative Research Incentives Scheme (VENI) under grant no. [275–53‐014], and by the ERC with the Advanced Grant (led by Prof. Bas van Bavel) under grant no. [339647]. The authors thank the “Coordinating for Life” ERC‐seminar group of Bas van Bavel, Bram van Besouw, Jessica Dijkman, Maïka de Keyzer, Vincent Schippers, and Matthew Hannaford for comments and suggestions. The authors also thank the anonymous referees for their comments which greatly helped to improve the article and Jonathan de Bruin for checking all calculations.

## Supporting information

Supporting Appendix 1

## Notes

1 This article follows the convention of referring to the first plague of the Second Pandemic that occurred in the period 1347–52 as “the Black Death” and all other later medieval plagues from that point onwards as recurring plagues; in line with the recent Alfani & Murphy, 2017.

2 We use mortmain accounts from Archives Départementales du Nord, Lille, B 12122–12226; National Archives of Belgium, Brussels, I, 004, 17867–73.

3 A sex ratio of 1:1 is equal parity in proportion of males and females dead.

4 These were Froidchapelle‐Fourbechies, Rousies, Montbliart, Solre‐Saint‐Géry, Villers, Vaulx, Leval‐sous‐Beaumont, Acren‐Saint‐Martin, Rance, Taisnieres, and Vieux‐Condé.

5 Because there are no issues with male‐female exemption, the full database of 25,610 deaths is used in Figure 1. The choice of over three average mortalities per locality is partially arbitrary, but represents the major spikes seen.

6 In the period 1420–3, evidence for plague was found in the Northern Netherlands, but none was forthcoming for the Southern Netherlands; see Supporting Information Appendix 1.

7 Because there are no issues with male–female exemption, the full database of 25,610 deaths is used in Figure 2.

8 We do not use localities that appear in every single year between 1395 and 1407 as that would create a sample size that is too small. Additionally we must acknowledge that many of these localities not present for a couple of the years between 1395 and 1407 is down to the fact that they had no mortalities to record in those years, rather than “disappearing” from the record, since they were often small hamlets.
